# Efficacy and Safety of Navitor Versus Sapien Valves in Transcatheter Aortic Valve Implantation: A Systematic Review and Meta-Analysis

**DOI:** 10.7759/cureus.110349

**Published:** 2026-06-06

**Authors:** Yusuf Ahmed, Abdulla Almubarak, Manar Ali, Jaafar Alsadeq Laith, Lujayn Juma, Walaa Yusuf, Zahraa Salman, Hana Asser, Mohamed Gabr, Ahmed Emara

**Affiliations:** 1 Department of Internal Medicine, Salmaniya Medical Complex, Manama, BHR; 2 Faculty of Medicine, Mansoura University, Mansoura, EGY; 3 Department of Ophthalmology, Dr. Haifa Eye Hospital, Manama, BHR; 4 Department of Cardiothoracic Surgery, Faculty of Medicine, Mansoura University, Mansoura, EGY; 5 Department of Cardiology, Faculty of Medicine, Menoufia University, Shebin El Kom, EGY

**Keywords:** balloon-expanding valves, heart valve, meta-analysis, navitor, sapien, self-expanding valves, systematic review, tavi, tavr

## Abstract

Transcatheter aortic valve implantation (TAVI) has become an established treatment option for symptomatic severe aortic stenosis, particularly in older patients. In this meta-analysis, we compare the latest generations of self-expandable valves, Navitor (Abbott Structural Heart, Santa Clara, CA, USA), with balloon-expandable valves, Sapien (Edwards Lifesciences, Irvine, CA, USA), in TAVI. We systematically searched PubMed, Scopus, Web of Science, and Cochrane Library databases. Studies were considered eligible if they compared Navitor with Sapien valves in TAVI. Our search strategy yielded four studies with a total of 4,828 patients. In terms of safety and efficacy, no statistically significant differences were noted between the two groups in device and technical success, 30-day all-cause mortality, stroke incidence, or major vascular complications. Permanent pacemaker implantation (PPI) (risk ratio (RR) 2.16; 95% CI 1.67 to 2.80; *P* < 0.001) and moderate-to-severe paravalvular leakage (PVL) (RR 3.36; 95% CI 1.93 to 5.82; *P* < 0.001) rates were significantly higher in Navitor valve patients. In contrast, Navitor was associated with lower rates of moderate-to-severe patient-prosthesis mismatch (PPM) (RR 0.55; 95% CI 0.31 to 0.98; *P* = 0.043) and better hemodynamic outcomes compared to Sapien. In summary, both Navitor and Sapien valves were effective and safe in TAVI. However, the Navitor valve was associated with higher rates of moderate-to-severe PVL and PPI. On the other hand, Navitor had a better hemodynamic profile and lower moderate-to-severe PPM than Sapien.

## Introduction and background

Aortic valve stenosis is the most common valvular heart disease in the Western world [1]. Transcatheter aortic valve implantation (TAVI) is a less invasive therapeutic procedure for aortic stenosis compared to conventional aortic valve replacement surgeries [2]. It emerged as the preferred treatment for symptomatic severe trileaflet aortic stenosis in patients aged ≥70 years with favorable anatomy according to the European Society of Cardiology and the European Association for Cardio-Thoracic Surgery guidelines, which consider it a Class IA recommendation [3]. On the other hand, the American College of Cardiology/American Heart Association guidelines recommend TAVI for symptomatic patients with severe aortic stenosis who are >80 years of age or for younger patients with a life expectancy <10 years and no anatomic contraindication to transfemoral TAVI. It is categorized as a Class IA recommendation [4]. However, paravalvular leakage (PVL) remains a main concern following TAVI, regardless of the surgical risk and valve type [5]. Thus, attention is progressively shifting toward the impact of the anatomical and technical factors on the procedural outcomes [6].

TAVI prostheses can be categorized into balloon-expandable valves, which are expanded via balloon inflation, and self-expandable valves, which are opened spontaneously and return to their predetermined dimensions once they are released within the vessel [2,7]. The latest generations of balloon-expandable valves include Sapien 3 Ultra and Sapien 3 Ultra Resilia (Edwards Lifesciences, Irvine, CA, USA), which are the fourth and fifth generations, respectively [8,9]. Sapien 3 Ultra has the same frame and leaflet design as Sapien 3, with the addition of an enhanced outer sealing skirt [10]. The latest generation of balloon-expandable valves, Sapien 3 Ultra Resilia, incorporates two important features: the Ultra skirt, which is designed to minimize PVL, and Resilia tissue that is characterized by anti-calcification activity that preserves the durability of the valve [11].

More recently, the Navitor valve (Abbott Structural Heart, Santa Clara, CA, USA), the latest generation of intra-annular self-expandable valves, reported favorable outcomes with design modifications mitigating the risk of PVL by featuring NaviSeal technology [12]. Despite the FDA approval and the widespread use of both valves, no randomized controlled trials (RCTs) have compared the Navitor valve to the Sapien valve. This head-to-head comparison is essential in order to distinguish between the clinical outcomes of both valves, rather than depending on the theoretical mechanisms and the expected and estimated outcomes, especially for their mechanisms in minimizing the risk of PVL.

To the best of our knowledge, this is the first systematic review and meta-analysis to assess the self-expanding Navitor valve and compare it to the balloon-expandable Sapien valves in terms of procedural, hemodynamic, and clinical outcomes in patients undergoing TAVI. This study gives an opportunity to individualize the valve choice. The decision for the appropriate valve will be based on the patient’s situation and the benefit-to-risk ratio.

## Review

Methods

This systematic review and meta-analysis were conducted in accordance with the PRISMA guidelines [13]. This study is registered in the International Prospective Register of Systematic Reviews (PROSPERO) under the protocol (CRD420251101858).

Study Eligibility

Studies were deemed eligible if they met the following inclusion criteria: (1) RCTs and observational studies, including retrospective and prospective cohort studies; (2) comparing Navitor versus the latest generations of Sapien valves (Sapien 3 Ultra and Sapien 3 Ultra Resilia); and (3) patients aged 18 years or older. Studies were excluded if (1) not published in English; (2) meta-analysis and review articles; and (3) overlapping study populations.

Search Strategy

PubMed, Scopus, Cochrane Library, and Web of Science databases were systematically searched from inception until August 22, 2025, using the following search strategy (Navitor OR "Navitor valve" OR "Abbott Navitor" OR "Navitor TAVI" OR "Navitor THV" OR "Navitor transcatheter heart valve" OR "self expandable" OR SEV) AND ("Sapien 3" OR "Sapien 3 Ultra" OR "Sapien 3 Ultra Resilia" OR "balloon expandable" OR "Edwards Sapien 3" OR BEV). The same Boolean search strategy was applied across all databases. The number of records retrieved from each database was as follows: PubMed (174), Scopus (276), Cochrane Library (44), and Web of Science (187). In addition, gray literature, conference abstracts, and trial registries were not systematically searched.

Screening Process

Two authors (YA and MA) independently screened titles and abstracts according to the prespecified eligibility criteria. Full-text screening of potentially eligible studies was subsequently performed by the same authors. Screening and duplicate removal were conducted using Zotero software. Any disagreements during screening were resolved through panel discussion with AE and MG.

Data Extraction

Two authors (YA and MA) independently extracted data according to the prespecified eligibility criteria. Any disagreements during data extraction were resolved through a panel discussion with AE and MG. It was verified that all the included studies are free from overlap.

Outcomes Definitions

Outcomes extracted included device and technical success, in-hospital and 30-day all-cause mortality, incidence of stroke, permanent pacemaker implantation (PPI), major vascular complications, valve-in-valve interventions, PVL, patient-prosthesis mismatch (PPM), mean aortic pressure gradient, effective orifice area (EOA), and index EOA. All clinical outcomes and hemodynamic parameters were defined and graded according to the Valve Academic Research Consortium 3 consensus criteria [14].

Quality Assessment

The Risk of Bias in Non-randomized Studies of Interventions (ROBINS-I) tool was utilized to assess the included studies of this meta-analysis [15]. The ROBINS-I tool addresses the risk of bias of observational studies based on the following seven domains: bias due to confounding factors, selection of participants, classification of interventions, deviations from intended interventions, missing data, measurement of outcomes, and selection of the reported results. An overall risk of bias judgment was reached based on the seven assessed domains. The assessment was performed independently by two authors (YA and WY). Any disagreements during risk-of-bias assessment were resolved through a panel discussion with AE and MG.

Statistical Analysis

Continuous outcomes included in the analysis were compared by mean difference (MD) and 95% CIs with a random effect model. Binary outcomes were assessed using risk ratio (RR) and 95% CIs using the random-effects model (Mantel-Haenszel statistical method). Between-study variance was estimated using restricted maximum likelihood for continuous outcomes and the DerSimonian-Laird method for binary outcomes. Endpoints were considered statistically significant if the p-value was less than 0.05. Leave-one-out sensitivity and subgroup analysis based on Sapien variants were utilized to explore heterogeneity of studies with moderate-to-high heterogeneity. Statistical analysis of this study was conducted using the meta package in R version 4.5.0 (R Foundation for Statistical Computing, Vienna, Austria).

Results

Study Selection and Characteristics

**Figure 1:**
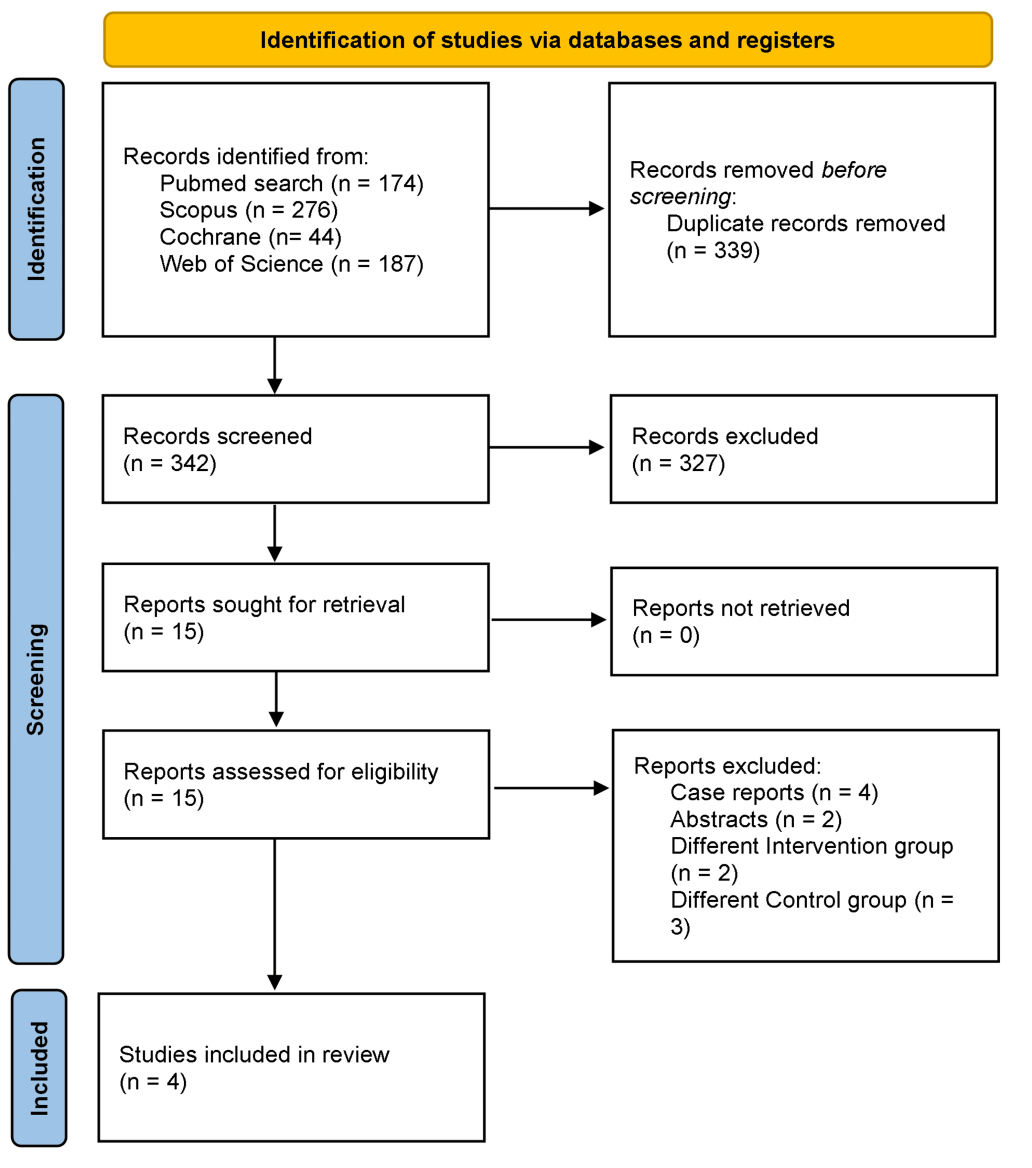
PRISMA flow diagram of study screening and selection

Our search strategy yielded 681 potential studies from PubMed, Scopus, Cochrane Library, and Web of Science, as illustrated in Figure 1. After removing duplicates and studies not meeting the inclusion criteria based on title and abstract (339 and 327 studies, respectively), 15 articles remained. They were assessed for inclusion based on the full text. A total of four studies were included for the quantitative analysis of this meta-analysis.

The baseline characteristics of the Navitor and Sapien groups are summarized in Table 1. 

**Table 1:** Baseline characteristics of Navitor/Sapien EuroScore II: European System for Cardiac Operative Risk Evaluation II; LVEF: left ventricular ejection fraction; STS: Society of Cardiothoracic; STS PROM: Society of Cardiothoracic Predicted Risk of Mortality; VARC: Valve Academic Research Consortium

Author and year	Study type	Country	Intervention	Control	Patients (N)	Females, N (%)	Age (years), mean (SD)	LVEF %, mean (SD)	Mean aortic valve gradient (mmHg), mean (SD)	Aortic valve annulus area (mm²), mean (SD)	Surgical risk score (type, score), mean (SD)	Follow-up time	Follow-up completeness	VARC version
Eckel et al. (2025) [6]	Retrospective cohort	Germany	Navitor	Sapien 3 Ultra	104/75	91 (87.50%)/63 (84%)	83 (6.01)/78.94 (9.07)	60.01 (6.92)/58.76 (6.80)	39.65 (14.28)/44.34 (10.43)	392.18 (28.94)/396.30 (32.88)	EuroSCORE II 3.62 (2.18)/EuroSCORE II 3.02 (1.74)	30 days	Complete	VARC-3
Cannata et al. (2025) [7]	Retrospective cohort	Europe/United States	Navitor	Sapien 3 Ultra	1,746/2,176	1006 (57.62%)/1001 (46.00%)	81 (6.20)/80 (7.30)	55.33 (10.05)/56.09 (11.33)	47.36 (13.34)/45.61 (14.04)	430 (71)/444 (71)	STS PROM 4.28 (3.08)/STS PROM 3.89 (2.97)	One year	Incomplete	VARC-3
Iwata et al. (2025) [11]	Retrospective cohort	Japan	Navitor	Sapien 3 Ultra Resilia	44/53	31 (70.50%)/37 (69.80%)	86.65 (3.83)/84.65 (6.86)	66.57 (7.80)/63.87 (7.62)	46.42 (18.39)/44.18 (14.48)	359.54 (67.44)/402.20 (70.87)	STS SCORE 4.88 (2.38)/STS SCORE 5.94 (4.04)	30 days	Complete	VARC-3
Mizutani et al. (2026) [16]	Retrospective cohort	Japan	Navitor	Sapien 3 Ultra Resilia	315/315	267 (84.80%)/270 (85.70%)	85.65 (5.21)/85.65 (5.21)	64.65 (6.70)/64.65 (6.70)	45.10 (14.89)/43.70 (13.41)	369.25 (42.45)/364.70 (41.71)	STS PROM 6.30 (3.87)/STS PROM 6.31 (3.43)	Post-procedural	Incomplete	VARC-3

Valves Efficacy

**Figure 2:**
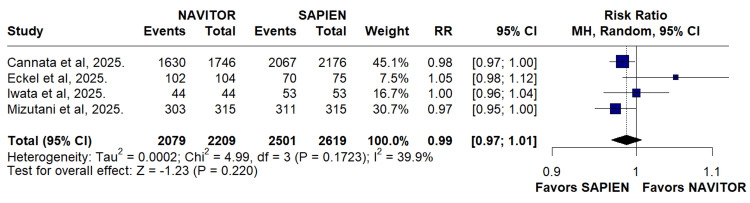
Technical success [6,7,11,16] MH: Mantel-Haenszel; RR: risk ratio

**Figure 3:**
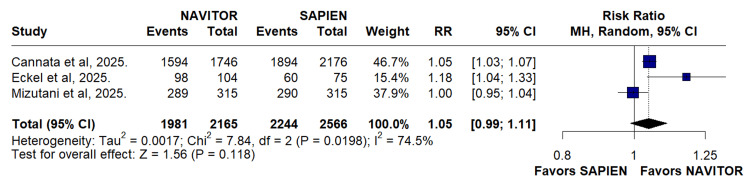
Device success [6,7,16] MH: Mantel-Haenszel; RR: risk ratio

In terms of valve efficacy, no statistically significant differences were observed when Navitor was compared to the Sapien group in terms of technical (RR 0.99; 95% CI 0.97 to 1.01; P = 0.220; I² = 39.9%; Figure 2) and device success (RR 1.05; 95% CI 0.99 to 1.11; P = 0.118; I² = 74.5%; Figure 3).

Mortality

**Figure 4:**
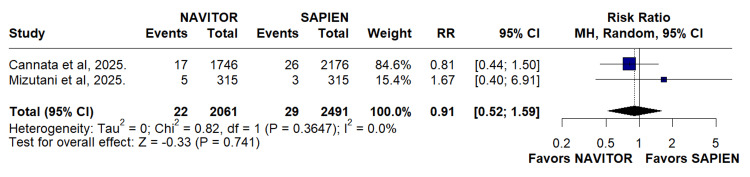
In-hospital mortality rates [7,16] MH: Mantel-Haenszel; RR: risk ratio

**Figure 5:**
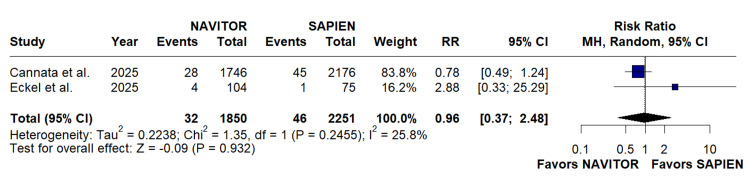
All-cause mortality at 30 days [6,7] MH: Mantel-Haenszel; RR: risk ratio

For mortality outcomes, both valves showed low rates of both in-hospital mortality (RR 0.91; 95% CI 0.52 to 1.59; P = 0.741; I² = 0%; Figure 4) and 30-day all-cause mortality (RR 0.96; 95% CI 0.37 to 2.48; P = 0.932; I² = 25.8%; Figure 5) with no statistically significant difference between the two groups.

Complications

**Figure 6:**
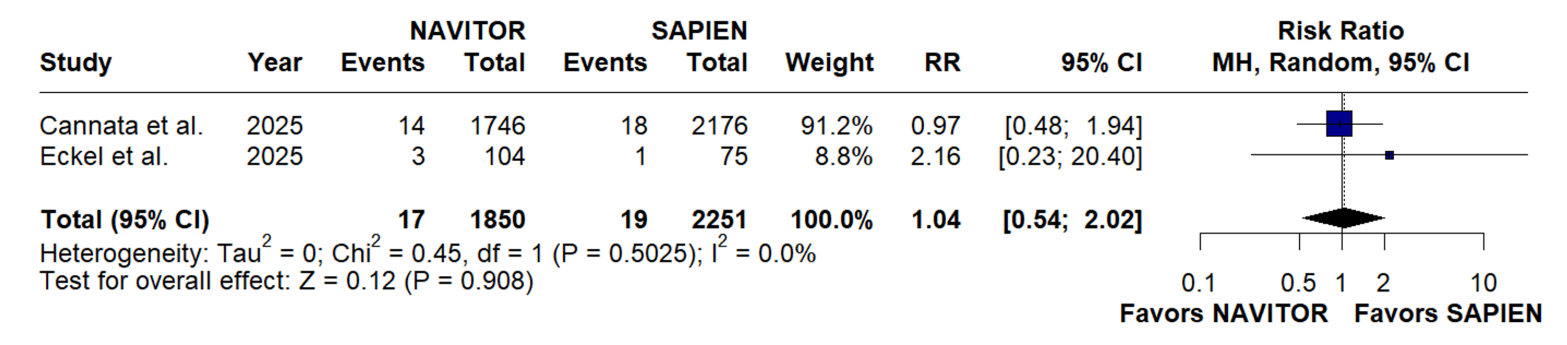
Incidence of disabling stroke [6,7] MH: Mantel-Haenszel; RR: risk ratio

**Figure 7:**
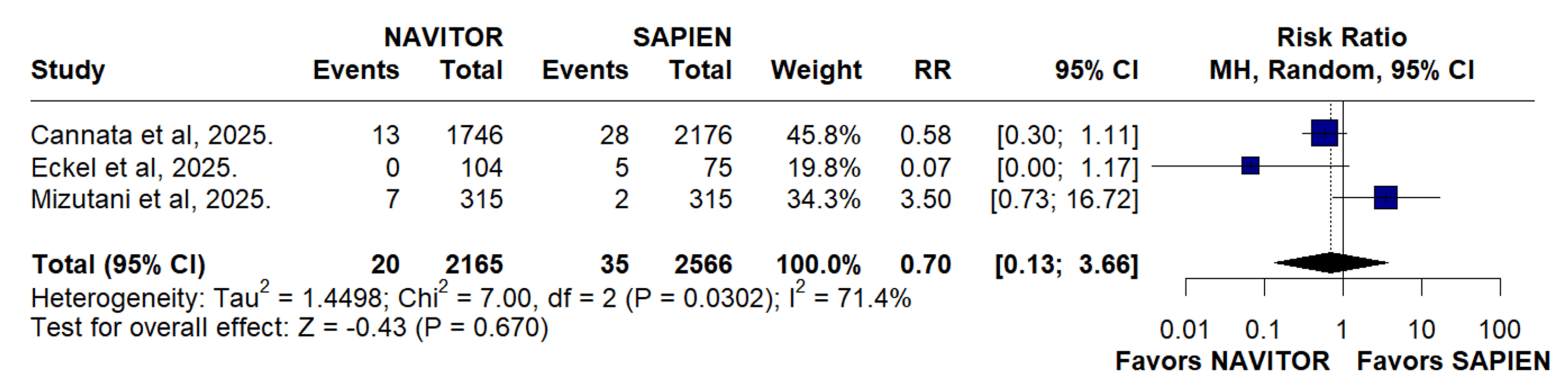
Incidence of major vascular complications [6,7,16] MH: Mantel-Haenszel; RR: risk ratio

**Figure 8:**
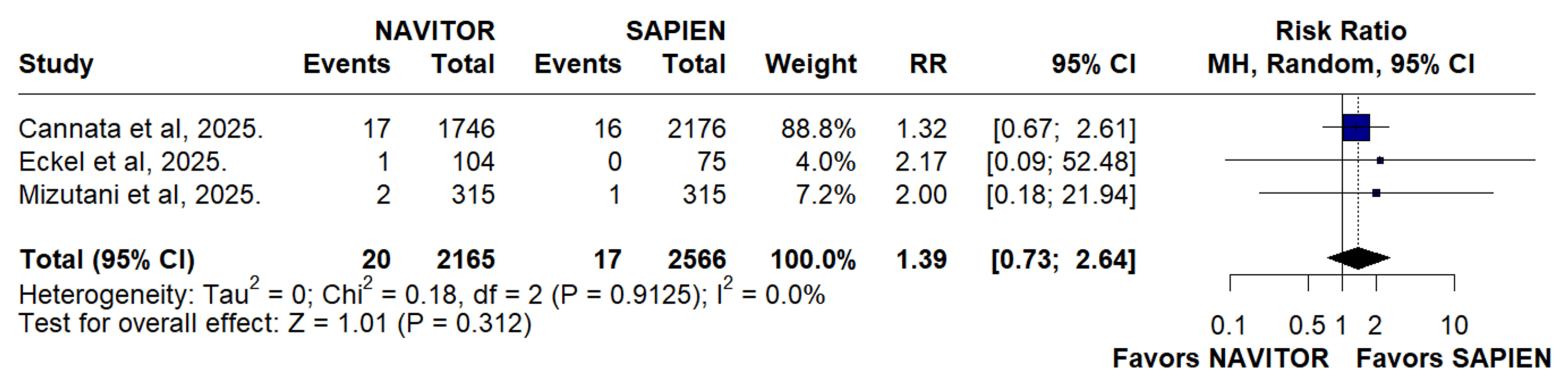
Incidence of valve-in-valve interventions [6,7,16] MH: Mantel-Haenszel; RR: risk ratio

**Figure 9:**
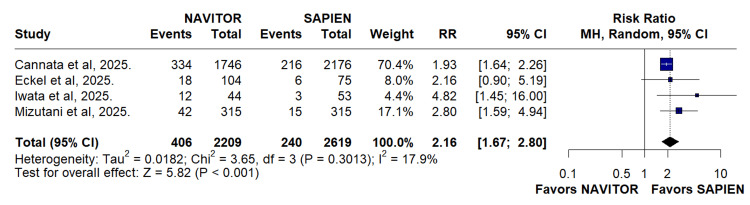
PPI [6,7,11,16] MH: Mantel-Haenszel; PPI: permanent pacemaker implantation; RR: risk ratio

In the analysis of complications following Navitor and Sapien 3 valves implantation, disabling stroke carried similar rates among both arms (RR 1.04; 95% CI 0.54 to 2.02; P = 0.908; I² = 0%; Figure 6). There was no statistically significant difference between Navitor and Sapien 3 in terms of major vascular complications (RR 0.70; 95% CI 0.13 to 3.66; P = 0.670; I² = 71.4%; Figure 7) and valve-in-valve interventions (RR 1.39; 95% CI 0.73 to 2.64; P = 0.312; I² = 0%; Figure 8). In the pooled analysis of PPI, the forest plot illustrated that patients who underwent TAVI using the Navitor valve had a higher need for PPI (RR 2.16; 95% CI 1.67 to 2.80; P < 0.001; I² = 17.9%; Figure 9).

Echocardiographic Findings

**Figure 10:**
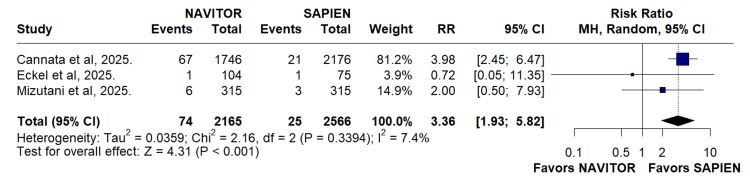
Moderate-to-severe PVL [6,7,16] MH: Mantel-Haenszel; PVL: paravalvular leakage; RR: risk ratio

**Figure 11:**
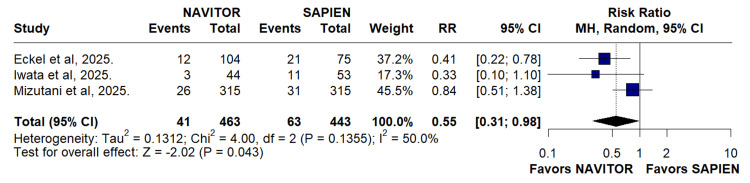
Moderate-to-severe PPM [6,11,16] MH: Mantel-Haenszel; PPM: patient-prosthesis mismatch; RR: risk ratio

**Figure 12:**
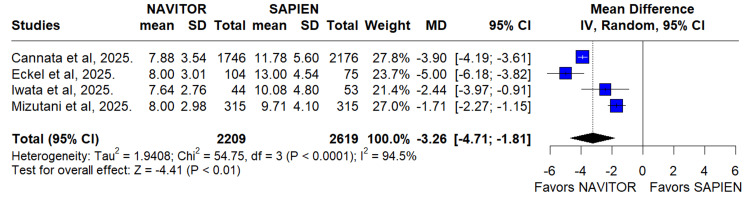
Mean aortic pressure gradient [6,7,11,16] IV: inverse variance; MD: mean difference

**Figure 13:**
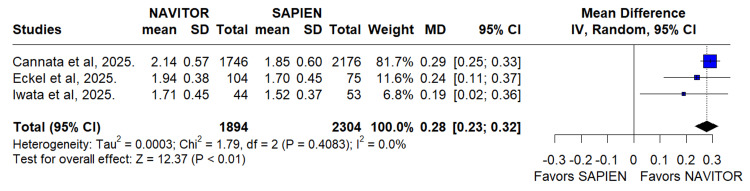
EOA [6,7,11] EOA: effective orifice area; IV: inverse variance; MD: mean difference

**Figure 14:**
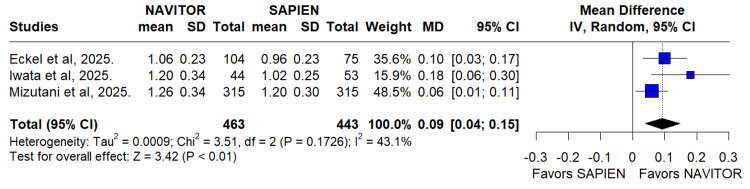
Index EOA [6,11,16] EOA: effective orifice area; IV: inverse variance; MD: mean difference

In terms of postprocedural echocardiographic findings, the Navitor valve had a higher rate of moderate-to-severe PVL (RR 3.36; 95% CI 1.93 to 5.82; P < 0.001; I² = 7.4%; Figure 10). In the pooled analysis of moderate-to-severe PPM, Navitor carried a lower rate when compared to Sapien (RR 0.55; 95% CI 0.31 to 0.98; P = 0.043; I² = 50.0%; Figure 11). In addition, the mean aortic pressure gradient was found to be significantly higher in patients who underwent TAVI by the Sapien valves (MD -3.26; 95% CI -4.71 to -1.81; P < 0.01; I² = 94.5%; Figure 12). EOA (MD 0.28; 95% CI 0.23 to 0.32; P < 0.01; I² = 0%; Figure 13) and index EOA (MD 0.09; 95% CI 0.04 to 0.15; P < 0.01; I² = 43.1%; Figure 14) were larger in the Navitor valve in comparison to the Sapien valves.

Sensitivity Analyses

**Figure 15:**
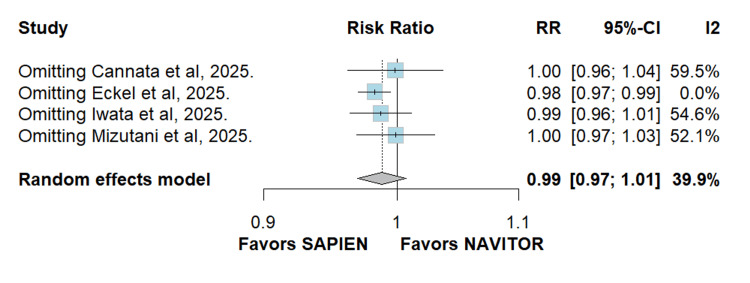
Leave-one-out sensitivity analysis of the outcome technical success [6,7,11,16] RR: risk ratio

**Figure 16:**
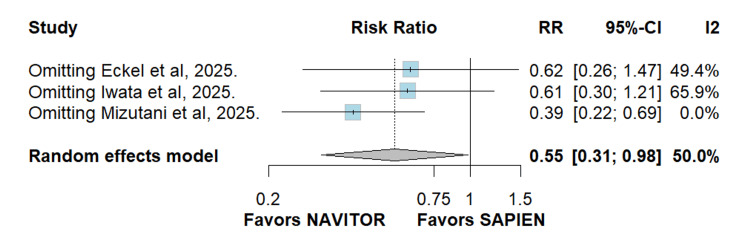
Leave-one-out sensitivity analysis of the outcome moderate-to-severe PPM [6,11,16] MD: mean difference; PPM: patient-prosthesis mismatch

**Figure 17:**
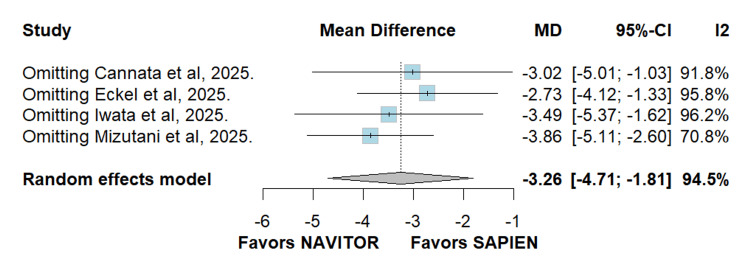
Leave-one-out sensitivity analysis of the outcome mean aortic pressure gradient [6,7,11,16] MD: mean difference

**Figure 18:**
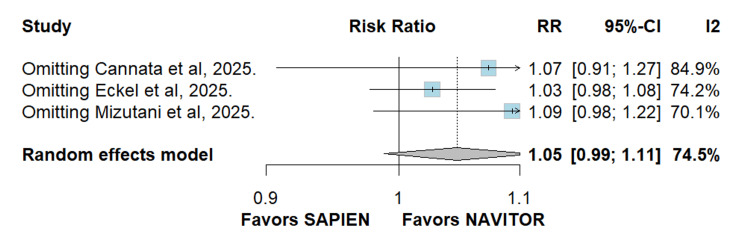
Leave-one-out sensitivity analysis of the outcome device success [6,7,16] RR: risk ratio

**Figure 19:**
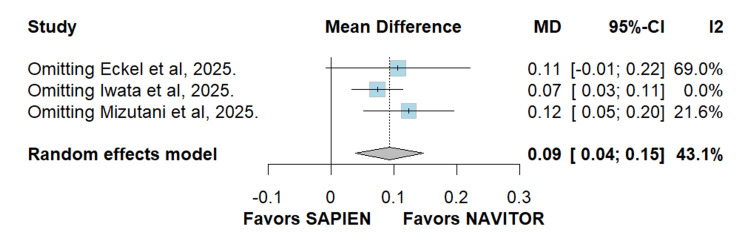
Leave-one-out sensitivity analysis of the outcome index EOA [6,11,16] EOA: effective orifice area; MD: mean difference

**Figure 20:**
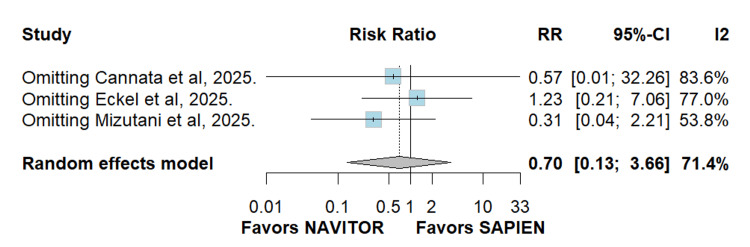
Leave-one-out sensitivity analysis of the outcome major vascular complications [6,7,16] RR: risk ratio

Leave-one-out sensitivity analysis was conducted for outcomes with moderate-to-high heterogeneity. Technical success showed statistical significance in favor of Sapien valves after omitting Eckel et al. [6], and heterogeneity was eliminated among the remaining three studies (I² = 0%; Figure 15). By excluding Mizutani et al. [16] from the outcome of moderate-to-severe PPM, the result remained statistically significant, and heterogeneity was eliminated (I² = 0%; Figure 16). For other endpoints with high heterogeneity, leave-one-out sensitivity analysis did not result in major changes in heterogeneity (Figure 17, Figure 18, Figure 19, Figure 20).

Subgroup Analyses

**Figure 21:**
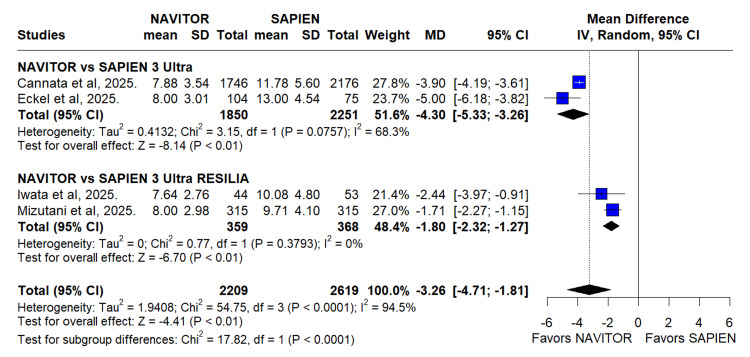
Subgroup analysis based on the Sapien valve variant for the outcome mean aortic pressure gradient [6,7,11,16] IV: inverse variance; MD: mean difference

**Figure 22:**
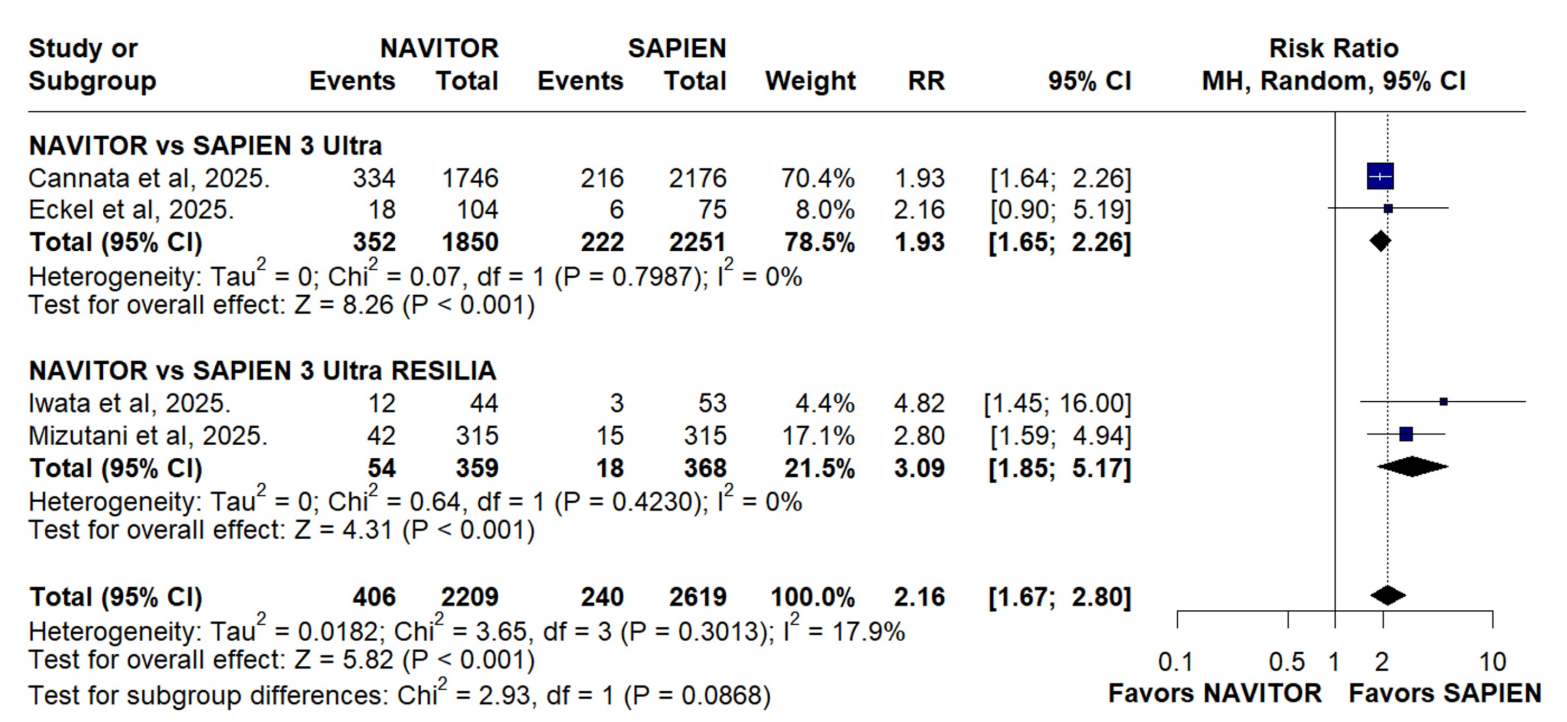
Subgroup analysis based on the Sapien valve variant for the outcome PPI [6,7,11,16] MH: Mantel-Haenszel; PPI: permanent pacemaker implantation; RR: risk ratio

**Figure 23:**
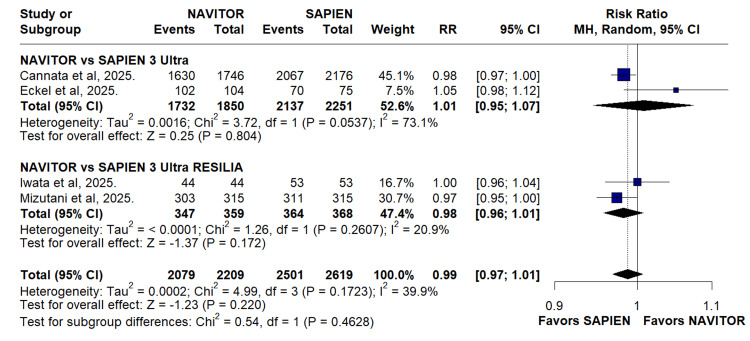
Subgroup analysis based on the Sapien valve variant for the outcome technical success [6,7,11,16] MH: Mantel-Haenszel; RR: risk ratio

Subgroup analysis illustrated that Navitor is superior to both Sapien 3 Ultra and Sapien 3 Ultra Resilia in terms of mean aortic pressure gradient by echocardiography (Figure 21). Regarding PPI, both Sapien variants showed a lower need for implantation post-procedurally by subgroup analysis (Figure 22). No difference was noted in technical success among Navitor and Sapien valve variants by subgroup analysis (Figure 23).

Quality Assessment

**Figure 24:**
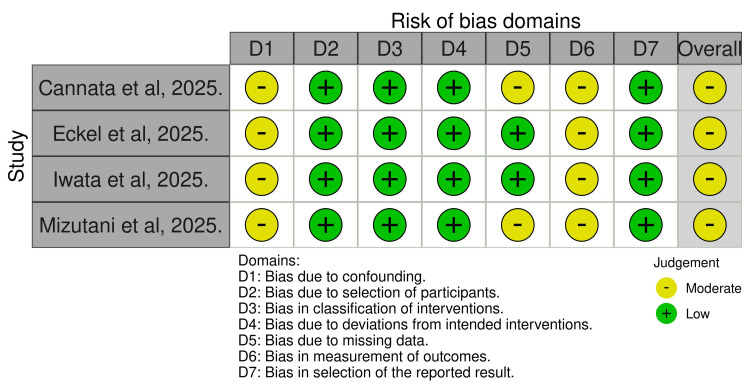
ROBINS-I of the included studies [6,7,11,16] ROBINS-I: Risk of Bias in Non-randomized Studies of Interventions

The risk of bias in the included studies was assessed using the ROBINS-I tool (Figure 24), which illustrated a moderate overall risk of bias across all included studies. This rating was predominantly driven by domain 1 (bias due to confounding), as these retrospective studies are susceptible to confounding by indication in the absence of randomization. To mitigate this risk, Cannata et al. [7] and Mizutani et al. [16] performed a propensity-score adjustment, which strengthens their internal validity. Despite its effective attenuation of bias from measured variables, it cannot account for unmeasured residual confounding. Additionally, domain 6 (bias in measurement of outcomes) has also contributed to this rating due to a lack of blinding.

Discussion

This is the first meta-analysis to compare Navitor and Sapien valves. The key findings were as follows: (1) The percentage of patients who needed PPI post-procedurally was shown to be higher in the Navitor group. (2) Echocardiographic findings of this study showed that the Navitor valve was favorable to the Sapien arm in terms of PPM, lower mean aortic pressure gradient, larger EOA, and index EOA. However, Sapien valves carried a lower rate of PVL. (3) There was no statistically significant difference noted in terms of in-hospital and 30-day all-cause mortality, incidence of disabling stroke, major vascular complications, and valve-in-valve interventions. (4) Both valves showed high efficacy, represented by the comparable device and technical success rates.

Navitor valve is the second generation of the self-expanding Portico valve (Abbott Structural Heart). The Navitor valve was enhanced by the inner and outer NaviSeal fabric cuffs to alleviate PVL by optimized sealing properties. Moreover, the FlexNav delivery system was improved to facilitate vascular access of small vessels up to 5 mm, which can lower vascular complications [17]. Regarding PVL, the pooled analysis of the included studies showed that Navitor has a higher rate of moderate-to-severe PVL when compared to Sapien valves (RR 3.36; 95% CI 1.93 to 5.82; P < 0.001; I² = 7.4%; Figure 10). The lower rates of moderate-to-severe PVL in the fourth and fifth generations of the Sapien valves are attributed to the textured polyethylene terephthalate skirt being 40% higher above the valve inflow compared to the third generation [18,19]. In contrast, a study by Eckel et al. [6] comparing Navitor to its predecessor, Portico, showed that Navitor carried a lower incidence of moderate-to-severe PVL (1.5% vs 7.2%; P < 0.041) [20]. This supports the role of the NaviSeal fabric cuffs to decrease rates of PVL, but Sapien 3 valves remain dominant in the reduction of PVL according to our pooled analysis [17]. An analysis of the combined PARTNER cohorts demonstrated an association between the presence of PVL and the increase in mortality rate, revealing the prognostic importance of PVL [21].

PPI is a serious postprocedural complication that raises mortality, prolongs hospital stay, and carries a high financial burden [22,23]. This meta-analysis showed that Navitor has a higher rate of PPI post-procedurally (RR 2.16; 95% CI 1.67 to 2.80; P < 0.001; I² = 17.9%; Figure 9). This was similarly noted when other self-expandable valves were compared to balloon-expandable valves [24-26]. Rates of PPI could be alleviated by adhering to the recommendations of Abbott, which sets 3 mm as the optimal implantation depth. Deeper implantation techniques were noted to have a higher need for PPI post-TAVI [27,28]. It is speculated that deeper implantations can compress the cardiac conduction system and lead to irreversible conduction abnormalities [29]. Additionally, the use of a 29 mm valve size and previous conduction abnormalities such as right bundle branch block and first-degree atrioventricular block were significantly associated with an increased need for PPI [27,28].

The one-year follow-up cohort of the NAVULTRA registry [7], which is one of the included studies in this systematic review and meta-analysis, showed that Navitor had a higher rate of mild PVL with no difference in moderate-to-severe PVL in comparison to Sapien 3 Ultra at one year of follow-up. On the other hand, Navitor valve patients still needed PPI compared to Sapien 3 Ultra [7]. In addition, the Navitor valve was used in low-to-intermediate risk patients in the Evaluation of Transcatheter Aortic Valve Replacement Using the Navitor Valve in a Global Investigation (VANTAGE) prospective single-arm study; no patients were reported to have moderate-to-severe PVL at 30 days and one year of follow-up. Moreover, low rates of all-cause mortality and disabling stroke were noted in the low-to-intermediate-risk population [30]. Similar findings were noted in the Portico NG prospective single-arm study that assessed the Navitor valve in high-risk patients at one year of follow-up [5]. This supports that the Navitor valve is safe and effective in TAVI of low-, intermediate-, and high-risk populations [5,30]. The Navitor valve was compared to the Evolut valves (Medtronic, Minneapolis, MN, USA), which are self-expandable valves. Navitor and Evolut valves showed similar hemodynamic outcomes, PVL, and device success. However, Evolut had a higher rate of disabling strokes at 30 days of follow-up [31].

The Navitor valve had a larger index EOA, which might have contributed to lower rates of PPM seen in our meta-analysis. Several factors are associated with a higher incidence of PPM post TAVI, including body surface area, valve-in-valve TAVI, balloon-expandable valves, and a smaller aortic annulus [32]. Smart trial findings showed that self-expandable valves are superior to balloon-expandable valves in patients with a small aortic annulus in terms of PPM; similar findings were noted in our study when Navitor and Sapien valves were compared [33]. PPM is a notable complication post-TAVI, as it leads to higher pressure gradients, affects the regression of left ventricular hypertrophy, and increases cardiac events and mortality rates, especially in severe PPM [34,35].

Limitations

There are several limitations to be acknowledged. All the included studies are retrospective and observational studies, carrying a high risk of bias; to alleviate the risk of bias due to confounding factors, propensity-matched score data were prioritized if available. Statistically, the limited number of studies (k = 2-4 per outcome) significantly reduced the power of heterogeneity tests and pooled stability assessments. It also contributed to the inability to perform publication bias tests, such as Egger’s regression or funnel plot. In addition, the findings for mean aortic gradient and device success should be interpreted cautiously due to high between-study heterogeneity despite sensitivity and subgroup analyses. Moreover, since the included studies were published in 2025, the results reflect a specific regulatory and learning-curve era, lacking temporal heterogeneity alongside a potential risk for overlapping populations across European multicenter registries.

Furthermore, operator and center experience remain a significant uncontrollable confounding, as the mature Sapien valve was compared to the newer Navitor valve. The short-term 30-day follow-up limited the assessment of durability, long-term valve thrombosis, and structural valve deterioration. Additionally, short-term echocardiographic gradients may not reflect the final performance, especially with a self-expanding frame like the Navitor. Thus, long-term head-to-head one-year echocardiogram data are needed to confirm these findings. A notable limitation is English-only exclusion criteria, which may introduce selection bias. It resulted in the omission of key Navitor data from the OCEAN-TAVI registry outputs and from the German/Italian center.

## Conclusions

Both Navitor and Sapien valves were effective and safe in TAVI. However, the choice of valve should be tailored to patients' characteristics. Sapien valves demonstrated a more favorable profile with respect to clinically relevant endpoints such as PPI and PVL, which would remain superior to Navitor valves, specifically in patients with previous conduction abnormalities. On the other hand, the use of Navitor might be more favorable in comparison to Sapien valves in specific populations like patients with a small aortic annulus, as Navitor provides a larger index EOA and lower mean aortic pressure gradient. Thus, this might mitigate the risk of PPM.
